# Enhancing scientific transparency in national CO_2_ emissions reports via satellite-based a posteriori estimates

**DOI:** 10.1038/s41598-023-42664-3

**Published:** 2023-09-18

**Authors:** Masataka Watanabe, Akihiro Oba, Yoko Saito, Gomboluudev Purevjav, Batjargal Gankhuyag, Munkhbat Byamba-Ochir, Batjargal Zamba, Tomohiro Shishime

**Affiliations:** 1 Research and Development Initiative, Chuo University, Tokyo, 1128551 Japan; 2 Information and Research Institute of Meteorology, Hydrology and Environment, Ulaanbaatar, 15160 Mongolia; 3 Climate Change Research and Cooperation Centre, Ministry of Environment and Tourism, Mongolia, Ulaanbaatar, 14191 Mongolia; 4 Graduate School of Science and Engineering, Chuo University, Tokyo, 1128551 Japan

**Keywords:** Climate change, Atmospheric science

## Abstract

Biennial Update Reports (BURs) are essential requirements from the United Nations Framework Convention on Climate Change (UNFCCC). However, many non-Annex I countries have not submitted these reports due to difficulties in compiling the inventories. We developed a satellite-based method for the top-down inverse estimation of CO_2_ emissions using partial-column data in the lower troposphere obtained by the Greenhouse Gases Observing Satellite, adopted to validate the Mongolian 2^nd^ BUR (BUR2) for the energy sector in 2018. The estimated CO_2_ emissions were only 1.5% higher than those reported in the BUR2; these were also very close (4.2% smaller) to estimates from the Emission Database for Global Atmospheric Research. Mongolia is the first country to introduce an independent inverse estimate in its BUR, thereby increasing scientific transparency. Our method could be applied into other countries and could be incorporated into UNFCCC reporting guidelines, significantly improving global CO_2_ emission estimates.

## Introduction

Anthropogenic CO_2_ is the most significant contributor to climate change. An increase in global temperature of more than 1.5–2 °C will occur during the twenty-first century unless a considerable reduction in CO_2_ and other greenhouse gas (GHG) emissions occurs in the coming decades^1^. National GHG inventories are an essential input to the Global Stocktake (GST), which was established in 2021 by the 26^th^ Conference of the Parties. The GST aims to assess the collective progress towards achieving the aims of the Paris Agreement and its long-term goals. All parties are required to submit a GHG inventory as part of the Biennial Transparency Report (BTR) under the Enhanced Transparency Framework^2,3^. Countries under the United Nations Framework Convention on Climate Change (UNFCCC) calculate CO_2_ emissions based on a bottom-up approach according to standard guidelines developed by the Intergovernmental Panel on Climate Change (IPCC)^4^. IPCC guidelines for National Greenhouse Gas inventories provide comprehensive information for each GHG emission and sink, including standard calculation methods along with parameters for standardized emission and absorption factors. Moreover, individual countries determine their own calculation methods, considering the actual domestic situation, data availability, and scientific knowledge of each emission and sink. However, such bottom-up approaches can lead to significant uncertainties in relation to missing social and economic information and inaccurate emission factors. In 2022 the National Academies of Sciences, Engineering, and Medicine published an assessment of the current capabilities of the inverse top-down, bottom-up, and hybrid approaches against the six evaluation pillars, including transparency, usability and timeliness, evaluation and validation, completeness, inclusivity, and communication^5^. However, important considerations regarding cost-effectiveness and less labour requirements were omitted.

Since satellite data and the inversion method are features of the top-down approach, cost-effective and less labour-intensive methods are advantages of the top-down approach compared with the bottom-up approach.

A comparison of the inverse top-down and bottom-up approaches indicated that transparency are difficult to assess. Nevertheless, the former has an overwhelming advantage because it is cost-effectiveness, less labour-intensive, and has favourable evaluation and validation^5^ features.

Approximately half of non-Annex I countries have not yet submitted Biennial Update Reports (BURs) to the UNFCCC due to knowledge and resource (financial and labour) gaps^6,7^, and practical solutions for shrinking these gaps are necessary. Specifically, we believe it is possible to shrink these knowledge and resource gaps by using a cost-effective, less labour-intensive, transparent, top-down approach with a great capacity for evaluation and validation, and making it possible for all non-Annex I countries to submit BTRs to the UNFCCC.

In practice, hybrid approaches have a strong usability that optimizes the integration of bottom-up and top-down approaches to provide users with the best available comprehensive GHG emissions information^5^. The 2019 Refinement of the IPCC 2006 report describes an independent approach based on atmospheric observations and inverse analysis (top-down approach) and recommends that bottom-up emission inventories be scientifically validated^8^. A successful GST is based on a submission of validated GHG inventory from all non-Annex I countries; the critical challenge is to accelerate these independent validation efforts, close the knowledge and resource gaps so that all participating countries can submit reports, and improve the transparency of emission inventories in each country. In this direction, an ambitious project was carried out in Mongolia, which entailed the use of position satellite-based inverse analysis results in validating the BUR. Providing information on knowledge and resources in the BTR will be a significant challenge. Here, considering Mongolia as a case study, we propose an a posteriori method that uses satellite data to verify CO_2_ emissions estimates contained in the BUR.

Mongolia plans to submit its 2^nd^ BUR (BUR2) with data through 2019. We propose including our estimates, obtained with a top-down approach, in BUR2. Mongolia has massive coal reserves^9^; thus, its primary energy source is coal^10^. In addition, Mongolia's national CO_2_ emissions for 2018^11^ were the highest in the energy, contributing to 97.8% of the total, followed by those relating to industrial processes and product use with 2.2%. CO_2_ emissions from the energy sector amount to 20.3 Tg. Furthermore, urban areas are responsible for approximately 70% of global fossil fuel-related emissions^12^ and play an essential role in mitigation strategies under the Paris Agreement's action plan. Consistently, emissions from the capital Ulaanbaatar account for approximately 69% of anthropogenic CO_2_ emissions in Mongolia (Supplementary Table S1). Therefore, we define Ulaanbaatar as our target city for obtaining CO_2_ emission estimates using the top-down approach.

**Figure 1:**
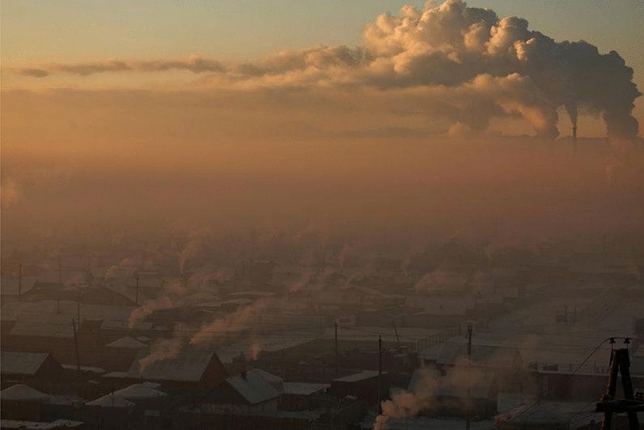
Typical winter scenery in Ulaanbaatar, Mongolia. *Copyright*: 2017 Reuters/B. Rentsendorj < https://static.reuters.com/resources/r/?m=02&d=20170207&t=2&i=1171734569&r=LYNXMPED160TP&w=1920 > (Thomson Reuters permitted the use of this photograph).

Approximately 60% of CO_2_ emissions in Ulaanbaatar originate from coal-fired power plants, 29% from coal stoves in “ger” (traditional transportable dwelling) districts, and 9% from automobiles^13^. Coal-fired power plants supply electricity and hot water to the urban area in the centre of the capital. Climate change has caused frequent mass livestock death events (called “dzud”) owing to summer droughts and heavy winter snowfalls (approximately 30% of all livestock deaths in Mongolia in 2010 were caused by dzud^14^). Dzud forced small herding families who lost their livestock to migrate with their gers and household goods around the capital city of Ulaanbaatar. Due to the Mongolian tradition of pastoralism, the government allowed people to occupy a specific portion of land anywhere in the country, resulting in the formation of sprawling ger settlements surrounding Ulaanbaatar (currently, migration is restricted by the government). In addition, the severe weather in winter, with temperature as low as − 40 °C, and the city’s location in a topographic depression that creates an atmospheric inversion layer, results in the trapping smoke from ger districts below the inversion layer, causing the worst air pollution in the world^15–17^, especially in the winter season (Fig. 1).

Our approach, targeting 2018, relies on a Bayesian framework and uses data from the Greenhouse Gases Observation Satellite (GOSAT) and a high spatial resolution regional atmospheric transport model^18–22^. A high-resolution regional atmospheric transport model is capable of capturing fine-scale variability in XCO2 distributions caused by transport and emission processes at urban scales^18^. Second, using the lower-layer concentrations from the GOSAT-based 2-layer analysis product by the Japan Aerospace Exploration Agency/Earth Observation Research Center (JAXA/EORC), the approach allows the quantification of an increase in observed CO_2_ concentrations within the city^23,24^.

We introduced independent science-based techniques to validate GHG emissions inventory reporting and contribute to scientific transparency^25^ in Mongolia. This approach could be applied to other countries and regions, integrated into the Paris Agreement's enhanced transparency framework, and may be proposed as a milestone in the UNFCCC’s inventory assessment for the GST.

## Results

### CO_2_ emissions from Ulaanbaatar

Three series of satellites observe the XCO_2_ concentration: the GOSAT series, the TanSat series, and the Orbiting Carbon Observatory (OCO) series. Among them, only the GOSAT series can observe the vertical distribution of the XCO_2_ concentration by sensing shortwave infrared (SWIR) and thermal infrared spectral band data based on the Fourier transform method. The lower and upper partial columns in the troposphere are identified according to the pressure ranges of 0.6–1 and 0.2–0.6 P_surf_, respectively, where P_surf_ is the pressure at the ground^23,24^. XCO_2_LT products are advantageous because they are not affected by thin clouds or aerosols^24^. For instance, XCO_2_LT data for Station 6 in the city centre of Ulaanbaatar are retrieved more than twice as frequently as National Institute for Environmental Studies (NIES) GOSAT v02.95-02.97 products. Here, we present the analysis results for anthropogenic CO_2_ emissions considering the atmospheric environment in the city of Ulaanbaatar using GOSAT-EORC-Daily-Partial-Column-GHG data from JAXA/EORC^26^.

**Figure 2:**
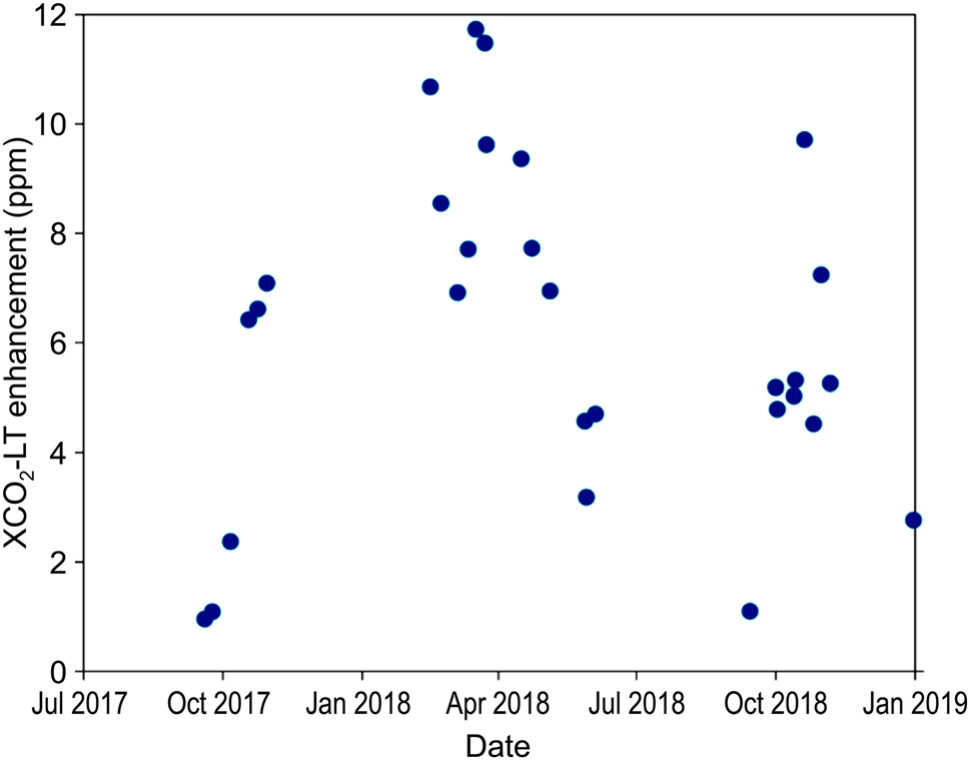
Area-averaged enhancement of XCO_2_LT in Ulaanbaatar. The enhancement (daily averaged) was calculated by subtracting the observed XCO_2_LT from the calculated monthly area-averaged XCO_2_UT.

A comparison between in-situ CO_2_ observations and the results of the WRF-Chem simulation is shown in Supplementary Fig. S1, Supplementary Table S2, and Supplementary Note 1. Figure 2 displays an observation time series of GOSAT in terms of the difference between the averaged XCO_2_ concentration in the lower troposphere (i.e., 0.6–1 P_surf_ in Ulaanbaatar; XCO_2_LT) and that in the upper troposphere (i.e., 0.2–0.6 P_surf_ in Ulaanbaatar; XCO_2_UT), which is defined as the XCO_2_LT enhancement in Ulaanbaatar. The XCO_2_UT concentration is calculated by taking monthly average area-averaged value data as a reference; therefore, the XCO_2_LT enhancement is not affected by annual CO_2_ increases or seasonal changes. Furthermore, in Ulaanbaatar, the maximum mixed-layer height, i.e., the height of a capping temperature inversion or statically stable layer of air, is approximately 2000 m on a typical clear summer day. In winter, a thick temperature inversion layer (> 500 m) covers a weak and thin mixed layer (< 300 m)^27^ because radiative cooling at night lowers the near-surface temperature, which is stratified in the absence of convection. Therefore, the actual boundary layer lies well below 4 km in altitude^24^. In addition, CO_2_ emissions from the chimneys of thermal power plants and the ger districts move and diffuse horizontally in the layer below 4 km (Fig. 1, Supplementary Fig. S2). As CO_2_ emissions from Ulaanbaatar remain confined to the lower troposphere, a typical condition in a high latitude basin area, XCO_2_LT enhancement data can be considered representative of the CO_2_ emissions from the city^24^ (Supplementary Note 2).

In this study, the model domain is set as slightly larger than the city size (50 km × 50 km and 0.6–1 P_surf_; Supplementary Note 3). Fourteen target observed data from GOSAT for XCO_2_LT with 10.5 km resolution cover the model domain almost completely. Thus, we assume that such data completely capture the CO_2_ emissions trapped in the urban dome. This is especially true in winter, when CO_2_ emissions are more easily quantifiable than in summer owing to the thick inversion layer confining the gas to lower altitudes, which contributes to the amount of available observed data from GOSAT. Therefore, there are more available data for winter than for summer.

Figure 2 shows the XCO_2_LT enhancement in Ulaanbaatar. GOSAT can observe not only points along the orbit but also target points. The trend between the summer of 2017 and winter of 2018, when GOSAT began targeted observations in Ulaanbaatar, shows that CO_2_ concentrations in the lower troposphere were consistently higher than those in the upper troposphere. This confirms that the XCO_2_LT enhancement is closely related to the emissions from the city, thereby reflecting its CO_2_ emissions. Furthermore, the XCO_2_LT enhancement, which became increasingly prominent from fall to winter/spring, shows a seasonal change with a decrease towards the beginning of summer, consistent with a trend of increased emissions in winter and decreased emissions in summer.

**Figure 3:**
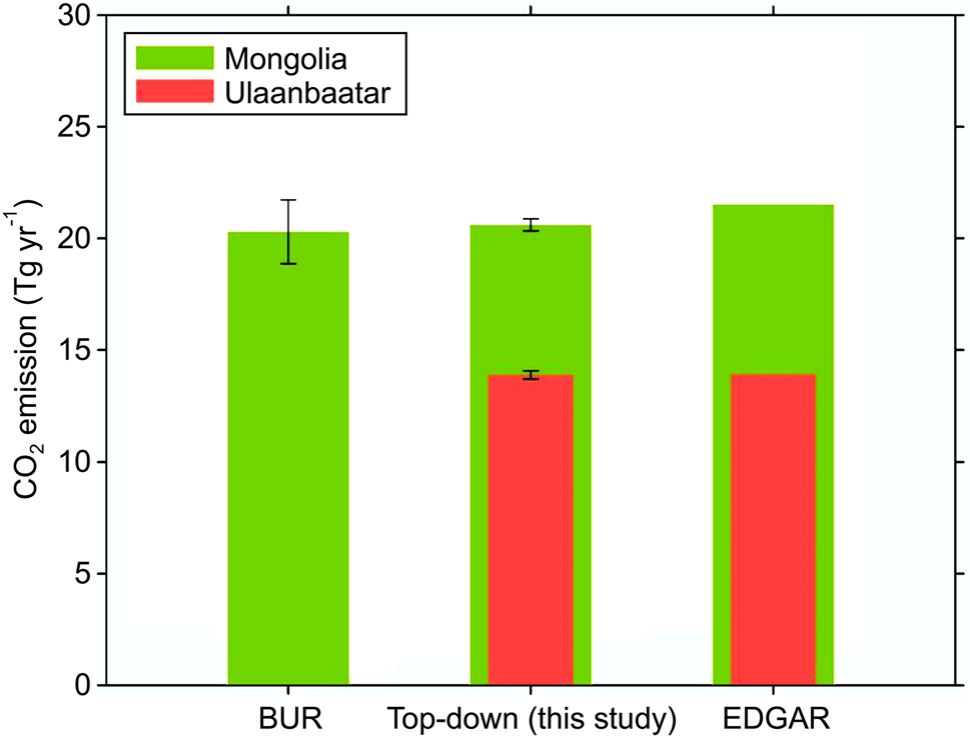
Comparison of Mongolia's bottom-up and top-down energy sector CO_2_ emissions in 2018. Mongolia's CO_2_ emissions (in Tg yr^−1^) for BUR, top-down estimates, and EDGAR. The vertical lines in BUR and top-down columns correspond to 95% confidence intervals.

Figure 3 shows the results of the inverse analysis of CO_2_ emissions in the energy sector in Ulaanbaatar using GOSAT satellite data with a top-down approach. We estimated the a posteriori CO_2_ emissions in the model domain surrounding Ulaanbaatar in 2018 using Green’s function between the CO_2_ concentration estimated by the atmospheric WRF-Chem transport model (see the comparison between modelled and observed XCO_2_ values in Supplementary Fig. S3) and 24 h-averaged XCO_2_LT values from GOSAT. In 2018, GOSAT conducted targeted observations in the model domain at 14 target observation points with a spatial resolution of 10.5 km. We only considered XCO_2_LT concentration data at times in which GOSAT successfully observed more than 10 target observation points. Finally, we obtained data for 23 days that we could use for the inverse analysis.

**Figure 4:**
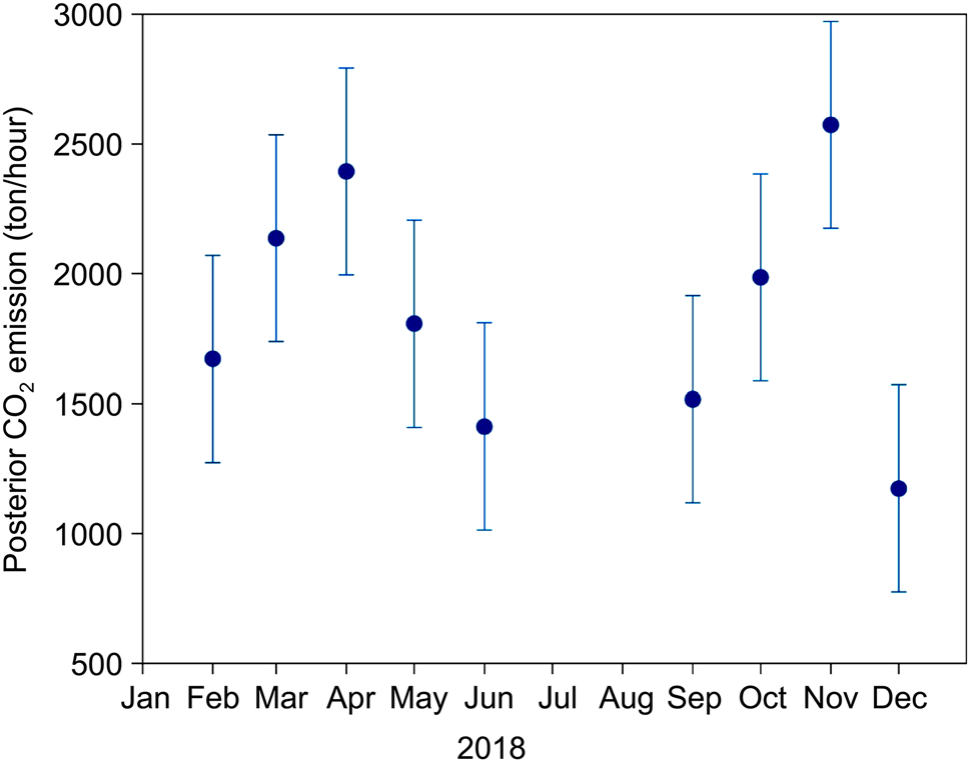
Inversion results of monthly-averaged CO_2_ emissions in Ulaanbaatar using GOSAT observation data in 2018. Each month other than June, September, November, and December features several data points. The vertical lines show the posterior uncertainties.

Green’s function requires a priori errors of GOSAT observations and CO_2_ emissions data as inputs. An observation uncertainty for XCO_2_LT concentration and the prior emission uncertainty is set to 2 ppmv and 400 ton h^−1^ (see Method). Additionally, a sensitivity analysis was performed on additional scenarios derived from different combinations with the observation and the prior emission uncertainties (Supplementary Table S3, Supplementary Note 4, and Supplementary Figs. S4–S15). Figure 4 shows the monthly-averaged CO_2_ emissions obtained from a posteriori CO_2_ emission estimates. The average percentage of a posteriori CO_2_ emissions to a priori CO_2_ emissions (the modification rate) is only 1.1% throughout the year (Supplementary Table S3). The posterior uncertainty of the scaling factor ranges from 0.12 to 0.34 (Supplementary Table S4), which is comparable to that evaluated in large cities such as Riyadh (0.17–0.24), Cairo (0.10–0.25), and Los Angeles (0.11–0.16)^18^. We derived a scaling factor (± 1σ uncertainty) for all observation days throughout the year (Supplementary Table S4), which was approximately 3%. Our result is consistent with the study of Ye et al. (2020)^18^ in that utilizing satellite observation over a longer time period potentially obtains emission estimates with lower uncertainty. This uncertainty underscores the value of integrating atmospheric constraints into our model, leading to more stable and reliable emissions estimates.

The a posteriori CO2 emissions estimates are only 0.2% higher than the value expected from a global emission inventory, the Emission Database for Global Atmospheric Research (EDGAR)^28^. In countries with limited data sources, such as Mongolia, there are cases in which BURs and National Inventory Reports use the same data sources as the global database to produce GHG inventories based on a bottom-up approach. Notably, although emissions in this study were estimated with a particular focus on the energy sector, a priori CO_2_ emissions and EDGAR values were already close to each other. Moreover, a posteriori CO_2_ emissions based on Green’s function reduce the gap to the EDGAR values even further.

### CO_2_ emissions in Mongolia

According to Mongolia's GHG inventory report, the CO_2_ emissions in 2014 were as follows: the energy sector accounted for 97.4% of total CO_2_ emissions, while industrial processes and product use sector, agriculture, forestry and other land use sector, and waste sector accounted for the remainder. There are no CO_2_ emission sources other than the energy sector in Ulaanbaatar. In the energy sector in Ulaanbaatar, the largest emissions are attributed to fuel combustion activities, accounting for 99.99%. Fuel combustion includes energy industries (electricity, heat, and other energy generation), manufacturing and construction industries, residential and commercial activities, and transport. Consequently, the top-down CO_2_ emission estimates consist of the energy sector. According to the percentage of CO_2_ emissions in Ulaanbaatar to those in the whole of Mongolia (Supplementary Table S1), we assume that this percentage did not change between 2014 and 2018 because economic growth in Mongolia during this period was minimal, partly owing to a conflict with the Mongolian government over taxation and financial agreements related to a foreign mining development project, which led to a pause in the project^29^. Thus, the economic structure of the capital city and rest of the country remained unchanged. Therefore, we consider that CO_2_ emissions from Ulaanbaatar account for 69% of CO_2_ emissions in Mongolia in our study.

The top-down CO_2_ emissions (20.6 ± 0.3 Tg yr^−1^) (all reported ranges are 95 percent confidence intervals) and energy sector CO_2_ emissions (20.3 ± 1.4 Tg yr^−1^) in BUR2 for 2018 differed by only 1.5% (0.3 Tg yr^−1^) (Fig. 3). The top-down CO_2_ emissions and energy sector emissions from EDGAR v6.0 differ by 4.2% (21.5 Tg yr^−1^). EDGAR v6.0’s energy sector CO_2_ emissions estimates were 6% higher than Mongolia’s energy sector CO_2_ emissions reported in BUR2. Therefore, the top-down approach presented in this study could reduce the difference between the estimate from the global CO_2_ emissions database and the value calculated in BUR2 in Mongolia.

## Discussion

We showed good agreement with the CO_2_ emissions of the energy sector computed in the upcoming BUR2 (with a discrepancy of only 1.5%). In addition, Mongolia’s a posteriori CO_2_ emission estimate was 4.2% smaller than that obtained from EDGAR v6.0^28^. The low uncertainty in our study can be primarily ascribed to the topographic features in Ulaanbaatar, as well as utilizing lower troposphere data with longer time from satellite observations, and applying detailed emission sources to the chimneys of thermal power plants and the ger districts in the atmospheric transport model.

### Hybrid Approaches

GOSAT satellites make observations covering an area with a 10.5 km diameter in a 160 km-wide grid. Therefore, there are many unobserved areas in Mongolia, and interpolation in those spaces becomes a problem. In this study, we conducted 14 intensive observations (with a diameter of 10.5 km) to cover the entire city of Ulaanbaatar, Mongolia. The use of an observation method that captures all CO_2_ emissions from Ulaanbaatar improves the accuracy of the observations. As such, the inverse analysis makes it possible to estimate the amount of CO_2_ emissions from Ulaanbaatar.

Regarding global fossil fuel emissions, one report states that urban areas account for approximately 70% of global fossil fuel emissions^12^ and play an essential role in mitigation strategies under the Paris Agreement's action plan. Ulaanbaatar is the largest city in Mongolia, and its fossil fuel-derived CO_2_ emissions account for 69% of the country’s total, which is most of the country's emissions. Notably, to prevent air pollution, population influx is restricted. Therefore, it is reasonable to assume that the emissions from Ulaanbaatar and Mongolia will remain unchanged^12^.

We establish that the inverse analysis accurately estimates 69% of fossil fuel-derived CO_2_ emissions in Mongolia. The remaining 31% can, thus, be obtained according to the ratio between the city and nationwide emissions rather than by inverse analysis. This is acceptable for Mongolia as it is a non-Annex I country with limited financial and human resources. “Hybrid Approaches,” combining the latest inverse analysis methods and all other applicable data analysis-based methods, have recently been recommended as a powerful means of reflecting estimated GHG emissions in the policy-making process more quickly^5^.

The Government of Mongolia decided to include the results of the National Emission Inventory 2018 by the “Hybrid Approaches” method described in Chapter 3, 3.1 *Inventory overview, Additional information/best practice* of the BUR2 to be submitted to the UNFCCC.

This approach is applicable in most, if not all, non-Annex I countries.

### Steps for further improving the method

The ratio between the city and nationwide emissions of fossil fuel-derived CO_2_ emissions could influence technological innovation towards decarbonization in each country in the future. To apply this ratio to countries other than Mongolia, it is necessary to upgrade the latest inverse analysis to a more general emission estimation method for a vast area that comprises not only urban areas but also an entire country. To this end, further development of future GHG observation satellite technology and improvement of the analysis system are necessary.

JAXA/NIES/MOEJ plans to launch the Next-Generation Greenhouse Gases Observing Satellite (GOSAT-GW) in 2024, it will make it possible to make planar observations over a swath of 911 km or more with a resolution of 10 km. Using these next-generation satellites, all areas, including many urban areas and areas with other land uses, will be covered with a 10 km resolution mesh. In addition, targeted observations such as those conducted in Ulaanbaatar in this research will be carried out in all other areas. With the advent of GOSAT-GW, it will become possible to uniformly apply the top-down method using satellite data to all land use areas. To incorporate GOSAT-GW, we are expanding and improving all analysis systems. We are preparing to apply the top-down method using satellite data in a unified manner to countries other than Mongolia.

Our approach is to first clarify the CO_2_ emissions from fossil fuel-based energy sources, which are Mongolia’s most important emission sources. However, there are various sinks and emission sources in Mongolia and other regions. Livestock is increasing yearly in Mongolia. Methane emissions from livestock accounted for 47.9% and 48.9% of the total emissions inventories in Mongolia in 2014 and 2018, respectively.

Observations covering a wide area can effectively detect methane emissions and CO_2_ absorption and emission from wide-ranging emission sources, such as grasslands and forest areas. Additionally, using GOSAT-GW in these areas, it is expected that a top-down method using satellites will be applicable to estimate the amount of CO_2_ absorption from grassland/forest areas and methane emissions from grazing livestock.

## Methods

### Methodology overview of a top-down approach to analyze nationwide CO2 emissions

Two approaches can be used for estimating GHG emissions: a bottom-up approach relying on forward analysis of global databases^28^ and top-down approach based on inverse analysis of observational data^29^.

Top-down approaches rely on inverse models such as Lagrangian particle models^31^ and Bayesian models based on Green’s function^32^. These models, however, have to contain and rely on a CO_2_ atmospheric transport model. These model can be a global transport model, such as the Goddard Earth Observing System model coupled to chemistry (GEOS-Chem)^33^, or a regional-scale model based on WRF-Chem^34^ (i.e., a regional model that relies on appropriately downscaled data extracted from a global transport model).

Ganesan et al.^30^ and Miller et al.^35^ reported examples of inverse analyses based on global transport models for India and China, respectively. In both study cases, methane emissions were analyzed by an inverse model using satellite data elaborated with GEOS-Chem and compared with BURs. In terms of CO_2_ inversion analysis, there have been studies such as Janarganan et al. (2020)^36^ who used a global model, and Zhang et al. (2021)^37^ who utilized a regional model. However, for most of non-Annex I countries like Mongolia, where observational data for CO_2_ emissions are limited, application of these analysis methods proves challenging. This is a common issue among non-Annex I countries in the UNFCCC, highlighting the need for developing a method that begins with the analysis at city level, where knowledge and observational data is relatively abundant such as air pollution monitoring data, regional weather model, and meteorological knowledge, which extends them to a nationwide estimation. Several examples of inverse analysis of CO_2_ emissions based on regional models targeting cities have been reported in the literature^18,19,38–40^. Pillai et al.^32^, for instance, used a 100-km square grid to estimate CO_2_ emissions in Berlin, Germany. Therefore, in this study, we first focus on a top-down approach to estimate CO2 emissions at the city level, specifically in Ulaanbaatar, Mongolia. This city-level data is then used as a basis for the nationwide CO_2_ emission estimation, creating a comprehensive picture of Mongolia's CO2 emissions.

### Inverse analysis of CO_2_ emissions in Ulaanbaatar

The process of estimating CO_2_ emissions consisted of three sequential steps (Supplementary Fig. S16): forward analysis using the regional chemical transport model WRF-Chem, inverse analysis using Green’s function, and comparison with a global database such as EDGAR.

#### Forward analysis

The spatial distribution of a priori CO_2_ emissions in Ulaanbaatar was estimated based on CO_2_ emission data from thermal power plants (four sites), automobiles, household stoves in gers, heat-only boilers, and coal-fired water heaters, which accounted for almost all the energy sector-related emissions in Mongolia in 2015. These data were estimated by the Japanese Ministry of Environment in the “Project on Development of Innovative Green Technology and MRV Method for JCM in Mongolia”, which was based on the same methodology as the Mongolian GHG emission estimate. Comparing the CO_2_ inventory for the energy sector in Mongolia from EDGAR with Mongolia's gross domestic product (GDP) for the same period, a constant ratio can be evaluated, suggesting that CO_2_ emissions in the energy sector are strongly related to the economic growth in Mongolia, and that no changes in economic structure occurred during the monitoring period. We calculated the a priori total CO_2_ emissions from Ulaanbaatar in 2018 according to this constant ratio with GDP^41^ (Supplementary Fig. S17). We calculated the hourly input data of a prior CO_2_ emissions for the forward model by differentiating observed data on hourly CO concentration changes provided by the Information and Research Institute of Meteorology, Hydrology and Environment in Mongolia (Supplementary Note 5, Supplementary Fig. S18).

We used the GHG Tracer developed by the Max Planck Institute and the National Oceanic and Atmospheric Administration as an atmospheric transport model for CO_2_. Consequently, we were able to sequentially analyse CO_2_ concentrations with a horizontal resolution of 9 km and 34 vertical levels. The modelled XCO_2_ was in good agreement with the GOSAT XCO_2_ data, with a correlation coefficient of 0.89. Supplementary Table S5 provides details on WRF-Chem physical conditions and main input parameters, including the modelling of the CO_2_ flux from vegetation (Vegetation Photosynthesis and Respiration Model, VPRM)^42,43^, and to Supplementary Fig. S3 shows the simulation results of XCO_2_.

#### Atmospheric transport model errors in forward analysis

Ye et al.^18^ estimated the error variance by combining the measurement error and forward model error variances. The former accounts for GOSAT measurements, whereas the latter considers transport model errors in wind speed, wind direction, and boundary conditions. WRF-Chem transport model uncertainties range within 0.31–3.03 ppm in Riyadh, Cairo, and Los Angeles^18^. In our model, we considered uncertainties in CO_2_ boundary conditions and the prior emissions.

Regarding boundary conditions, meteorological data and background CO_2_ concentration data—taken as boundary conditions in WRF-Chem—were obtained, from the final analysis data from the National Center for Environmental Prediction, USA^44^ and JENA CarboScope inversion system JENA_s04oc_v4.3 (shortened as JENA_s04oc)^45^ data from the Max Planck Institute, Germany. Although the resolution of JENA_s04oc was lower in our region (4° × 5°), the CO_2_ distribution was optimized using a Bayesian inversion framework^46,47^. The monthly average difference with the Atmospheric Infrared Sounder product of NASA’s AQUA satellite was approximately 1.1 ppmv in terms of column CO_2_ concentration in the Mongolian region (44–49 N, 100–110 E)^48^. In situ CO_2_ measurements by aircraft over Europe showed that JENA_s04oc products had a lower bias than those from the Copernicus Atmosphere Monitoring Service (CAMS)^49^, 0.8 (1.3) μmol mol^-1^ vs. 3.7 (1.5) μmol mol^−1^^50^, where the standard uncertainty in the final digits is given in brackets. This suggests that CAMS products are preferred for setting lateral boundary conditions for regional modelling. Consequently, we opted for JENA_s04oc products because the bias of CAMS in Mongolia was poorly constrained.

Regarding CO_2_ emissions, we quantified the discrepancy in CO_2_ concentrations between observed and model-predicted values (Supplementary Fig. S3).

#### Inverse analysis

We conducted the inverse analysis through a Bayesian inversion based on the synthesis inversion method (Green’s function method)^32^ and constructed Green's function to modify CO_2_ emissions to minimize the difference between the WRF-Chem simulated estimate and GOSAT observations by the following cost function J. This resulted in an a posteriori estimate of CO_2_ emissions.

1 $${\text{J}} = (c_{obs} - c_{fwd} - {\varvec{H}}_{x} )^{T} {\varvec{R}}^{ - 1} \left( {c_{obs} - c_{fwd} - {\varvec{H}}_{x} } \right) + \left( {x_{0} - x} \right)^{T} {\varvec{P}}_{0}^{ - 1} \left( {x_{0} - x} \right)$$

where c_obs_ is the observed CO_2_ mole fraction, c_fwd_ is the simulated CO_2_ mole fraction, H is Jacobi’s matrix (number of emission sources × number of observations per optimization period; e.g., 1 day), R is the c_obs_—c_fwd_ error covariance matrix, x is the modified amount of CO_2_ at the observation point, x_0_ is the prior emission estimate of x, and P_0_ is the error covariance matrix of x_0_.

In (1), the measurement error covariance matrix (diagonal matrix), R depends on the XCO_2_ random errors^32^ using GOSAT and TCCON observation data collected independently^51^. As a reference for R, Yoshida et al.^51^ showed that the standard deviation of the differences between XCO_2_ (SWIR L2 v02.xx product) and TCCON data are evaluated to be 2.1 ppm. Wang et al.^52^ also stated that using GOSAT data (ACOS dataset), setting 2 times (land) and 1.25 times (sea) of the presented retrieval error would give realistic errors. In (1), P0 represents the error covariance of the a priori CO_2_ emission and is defined as a diagonal matrix when the CO_2_ emission data as referred to by Pillai et al.^32^ are independently generated.

In this case, the optimal solution (a posteriori estimate of CO_2_ emissions) is given by:

2 $$\hat{x} = \left( {{\varvec{H}}^{T} {\varvec{R}}^{ - 1} {\varvec{H}} + {\varvec{P}}_{0}^{ - 1} } \right)^{ - 1} \left( {{\varvec{H}}^{T} {\varvec{R}}^{ - 1} \left( {C_{obs} - C_{fwd} } \right) + {\varvec{P}}_{0}^{ - 1} x_{0} } \right)$$

Jacobi’s matrix H^32^, which corresponds to Green's function, is a function of the impact of CO_2_ emissions from a given point on Earth's surface on the spatiotemporal variation in atmospheric concentrations. Therefore, forward modelling results based on an atmospheric transport model inputting pulsed emission fluxes or a Lagrangian analysis tracking the trajectories of CO_2_ gas can be used to populate H. The period during which emissions from the ground surface remain within the targeting area varies depending on the size of the targeting area and meteorological conditions (such as the wind speed). Therefore, we must formulate H for the period during which CO_2_ gas concentrations are recorded after the gas is emitted from within the model domain (Supplementary Fig. S19).

In R, Kuze et al.^24^ used a standard deviation of 2.09 ppm for both XCO_2_LT and XCO_2_UT because the value was validated by the TCCON network. Therefore, we also set a standard deviation of 2 ppm for our a priori error in the XCO_2_LT concentration data, which is also close to the value provided by the NIES^51^.

For P_0,_ Pillai et al.^32^ gave a 40% a priori emission error in CO_2_ emissions based on the difference between their detailed inventory data and the EDGAR inventory. Ye et al.^18^ chose an uncertainty of 20% for cities with low statistical uncertainty in CO_2_ emissions (Los Angeles) and 40% for cities with high statistical uncertainty (Riyadh, Cairo). In Ulaanbaatar, the statistical uncertainty is assumed to be high; however, the city is smaller than Riyadh and Cairo, and the number and location of primary sources of CO_2_ are known. Moreover, comparing the a priori CO_2_ emission data in this study with EDGAR energy sector data, the difference is as small as 1.3%. Therefore, we set the a priori emission uncertainty to 400 ton h^−1^ (26%).

In this study, Eqs. (1) and (2) were applied to a 50 km × 50 km model domain (refer to the Results, Supplementary Note 3, and Supplementary Fig. S20) in and around Ulaanbaatar in 2018 to calculate a posteriori estimates of GHG emissions, and XCO_2_LT concentrations were calculated from GOSAT/GOSAT-2 EORC Daily Partial Column GHG data^24,25^. CO_2_ emission values in Green's function corresponded to hourly-averaged emissions within the model domain over 24 h from the observation time of GOSAT. We calculated the a posteriori emissions only if 70% of the GOSAT observation points (10 out of 14 points) were available. Finally, we calculated the averaged ratio of a posteriori to a priori CO_2_ emissions within the model domain over the entire year.

After solving the cost function J, the following two formulas^18^ can be used to calculate an optimized estimate of the scaling factor and the a posteriori error variance:

3 $$\hat{\lambda } = \left( {y_{m}^{T} S_{o}^{ - 1} y_{m} + \sigma_{a}^{ - 2} } \right)^{ - 1} \left( {y_{m}^{T} S_{o}^{ - 1} y_{o} + \sigma_{a}^{ - 2} \lambda_{a} } \right){ }$$

4 $$\hat{\sigma }^{2} = \left( {y_{m}^{T} S_{o}^{ - 1} y_{m} + \sigma_{a}^{ - 2} } \right)^{ - 1}$$

where $\widehat{\lambda }=\widehat{x}$, y_m_ = H, S_o_ = R, and σ_a_=${{\varvec{P}}}_{0}$ based on (1) and (2).

### Comparison with a global database

Here, we describe the method to calculate a posteriori estimates of CO_2_ emissions for Ulaanbaatar and Mongolia.

First, we calculated the average ratio of a posteriori to a priori CO_2_ emissions at each GOSAT observation point. Second, we multiplied this ratio by the a priori estimate of energy sector CO_2_ emissions for Ulaanbaatar and Mongolia, thereby obtaining the a posteriori CO_2_ emissions of the energy sector. Finally, we calculated the 95% confidence interval (across all observation points during the whole of 2018) of the difference between a priori and a posteriori emissions at each observation point.

We calculated the a posteriori estimates of CO_2_ emissions in Mongolia using the following procedure. First, we calculated the a priori estimate of CO_2_ emissions in 2018 in Mongolia by multiplying the CO_2_ emissions in 2014 from the 1^st^ BUR in Mongolia by the 2018 GDP/2014 GDP^39^ ratio. Second, we calculated the a posteriori estimate of CO_2_ emissions in 2018 in Mongolia by multiplying the averaged ratio of a posteriori to a priori CO_2_ emissions within the model domain in Sect. 2 by the a priori estimate of CO_2_ in 2018 for Mongolia. Third, we calculated the difference between top-down and bottom-up CO_2_ emissions by calculating the difference between the a posteriori estimate of CO_2_ emissions and energy sector CO_2_ emissions in BUR2 for 2018. We used ArcGIS 10.2 (https://www.esri.com/en-us/arcgis/about-arcgis/overview) to extract subsets for Ulaanbaatar and Mongolia from the global CO_2_ emissions dataset in EDGAR v6.0. The latter contains energy sector CO_2_ emissions data for each administrative district. Finally, we calculated the difference between each subset and the a posteriori estimate of CO_2_ emissions.

### Supplementary Information

Supplementary Information.

## Data Availability

GOSAT XCO2LT data were obtained from https://www.eorc.jaxa.jp/GOSAT/CO2_monitor/index_Ver.K.html. Observed CO and CO_2_ data are available from the corresponding authors on reasonable request.
