# Supplementary material for: Enhancing scientific transparency in national CO2 emissions reports via satellite-based a posteriori estimates
Source: Sci Rep. 2023 Sep 18;13:15427. doi: 10.1038/s41598-023-42664-3 (PMC10507059; doi:10.1038/s41598-023-42664-3)
Supplement: Supplementary file 1 — Supplementary Information. [file 41598_2023_42664_MOESM1_ESM.pdf]

Enhancing scientific transparency in national CO_2 emissions reports via satellite-based a posteriori estimates

Masataka WATANABE[1*], Akihiro OBA[1], Yoko SAITO[1], Purevjav GOMBOLUUDEV[2], Gankhuyag BATJARGAL[2], Byamba-ochir MUNKHBAT[2], Zamba BATJARGAL[3], Tomohiro SHISHIME[4]

[1]Research and Development Initiative, Chuo University, Tokyo, 1128551, Japan; masawata@tamacc.chuo-u.ac.jp

[2]Information and Research Institute of Meteorology, Hydrology and Environment, Ulaanbaatar, 15160, Mongolia; p_gombo@hotmail.com

[3]Climate Change Research and Cooperation Centre, Ministry of Environment and Tourism, Mongolia, Ulaanbaatar, 14191, Mongolia; batjargal@ccrcc.mn

[4]Graduate School of Science and Engineering, Chuo University, Tokyo, 1128551, Japan; shishime.88f@g.chuo-u.ac.jp

* Corresponding author; email: masawata@tamacc.chuo-u.ac.jp

Supplementary Material

Supplementary Table S1: CO_2 emission estimates in Mongolia in the energy sector.

	2014	2015	2018
CO_2 emissions in the energy sector (Tg/yr)			
Ulaanbaatar City	11.61**	11.89*	13.73**
Mongolia emissions (BUR2)	17.23		20.29
GDP, million Tugrik (constant LCU) (World Bank National Accounts data and OECD National Accounts data)	22,362,588	22,894,781	26,446,671
Percentage of Ulaanbaatar CO_2 emissions in Mongolia (%)	67.39		68.68

*: Estimated by the governmental project

**: Estimated from GDP ratio with 2015 as the base year

Supplementary Table S2: Comparison of model performance on CO_2 (ppm) relative to observed CO_2 at 14:00 (local time) in 2018.

	Mean	Bias	SD*	RMSE
Observed	420.19		13.39	
Simulated	414.17	6.02	13.06	13.92

*SD: standard deviation

Supplementary Table S3: Inversion results of CO_2 emissions for different scenarios.

Date (yyyy/m/d, UTC)	A Std. error of a priori emissions: 200 ton/h Std. error of a priori XCO_2 observations: 2 ppmv				B Std. error of a priori emissions: 400 ton/h Std. error of a priori XCO_2 observations: 2 ppmv				C Std. error of a priori emissions: 800 ton/h, Std. error of a priori XCO_2 observations: 2 ppmv			
	GOSAT XCO_2 (ppmv)	Forward Model XCO_2 (ppmv)	A priori CO_2 emissions (ton/h)	A posteriori CO_2 emissions (ton/h)	GOSAT XCO_2 (ppmv)	Forward Model XCO_2 (ppmv)	A priori CO_2 emissions (ton/h)	A posteriori CO_2 emissions (ton/h)	GOSAT XCO_2 (ppmv)	Forward Model XCO_2 (ppmv)	A priori CO_2 emissions (ton/h)	A posteriori CO_2 emissions (ton/h)
2018/2/15 6:00	409.6	411.6	2293.0	2279.5	409.6	411.6	2293.0	2239.4	409.6	411.6	2293.0	2083.7
2018/2/22 6:00	407.4	411.5	1185.5	1165.2	407.4	411.5	1185.5	1104.7	407.4	411.5	1185.5	869.6
2018/3/5 6:00	413.6	414.3	2784.1	2775.7	413.6	414.3	2784.1	2750.6	413.6	414.3	2784.1	2653.0
2018/3/12 6:00	414.4	411.8	1420.0	1428.5	414.4	411.8	1420.0	1454.0	414.4	411.8	1420.0	1552.8
2018/3/17 6:00	418.4	412.4	2665.5	2686.7	418.4	412.4	2665.5	2750.0	418.4	412.4	2665.5	2995.8
2018/3/23 6:00	418.2	411.5	2045.0	2070.6	418.2	411.5	2045.0	2146.6	418.2	411.5	2045.0	2442.1
2018/3/24 6:00	416.3	411.1	1503.4	1523.4	416.3	411.1	1503.4	1582.9	416.3	411.1	1503.4	1814.3
2018/4/16 6:00	417.0	413.1	3479.1	3489.5	417.0	413.1	3479.1	3520.4	417.0	413.1	3479.1	3640.4
2018/4/23 6:00	415.4	413.2	1239.4	1246.6	415.4	413.2	1239.4	1268.0	415.4	413.2	1239.4	1351.2
2018/5/5 6:00	414.4	413.0	1160.8	1164.8	414.4	413.0	1160.8	1176.8	414.4	413.0	1160.8	1223.3
2018/5/28 6:00	412.1	411.8	2714.2	2710.2	412.1	411.8	2714.2	2698.1	412.1	411.8	2714.2	2651.4
2018/5/29 6:00	410.7	411.6	1577.2	1570.0	410.7	411.6	1577.2	1548.4	410.7	411.6	1577.2	1464.5
2018/6/4 6:00	411.6	410.5	1404.8	1406.7	411.6	410.5	1404.8	1412.3	411.6	410.5	1404.8	1434.2
2018/9/14 6:00	402.7	402.8	1531.8	1528.1	402.7	402.8	1531.8	1517.0	402.7	402.8	1531.8	1474.1

2018/10/1 6:00	409.2	405.9	3215.3	3223.7	409.2	405.9	3215.3	3248.6	409.2	405.9	3215.3	3345.4
2018/10/2 6:00	408.8	405.8	1290.8	1301.6	408.8	405.8	1290.8	1333.5	408.8	405.8	1290.8	1457.5
2018/10/13 6:00	409.0	407.3	2699.3	2701.5	409.0	407.3	2699.3	2708.2	409.0	407.3	2699.3	2734.3
2018/10/14 6:00	409.3	405.9	1228.9	1241.5	409.3	405.9	1228.9	1279.1	409.3	405.9	1228.9	1425.1
2018/10/20 6:00	413.7	407.0	1222.4	1249.6	413.7	407.0	1222.4	1330.3	413.7	407.0	1222.4	1644.0
2018/10/26 6:00	408.5	407.7	1346.1	1346.9	408.5	407.7	1346.1	1349.3	408.5	407.7	1346.1	1358.5
2018/10/31 6:00	411.2	408.0	2617.5	2626.4	411.2	408.0	2617.5	2652.9	411.2	408.0	2617.5	2755.8
2018/11/6 6:00	410.4	407.7	2545.8	2552.8	410.4	407.7	2545.8	2573.9	410.4	407.7	2545.8	2655.6
2018/12/31 6:00	415.3	414.3	1165.0	1167.2	415.3	414.3	1165.0	1173.8	415.3	414.3	1165.0	1199.3
Average			1927.6	1932.9			1927.6	1948.6			1927.6	2009.8
Averaged ratio of a posteriori CO_2 emissions to a priori CO_2 emissions (a posteriori/a priori CO_2 emissions×100-100)			0.3%				1.1%				4.3%	

Supplementary Table S4: Optimized CO_2 emission estimates for scenario B.

The values listed as 'scaling factor ± posterior uncertainty' in the top right corner represent the scaling factor and its corresponding posterior uncertainty (1σ) across all observation points.

Date (UTC)	**A priori CO_2 emissions (ton/h)**	**A posteriori CO_2 emissions (ton/h)**	Scaling factor ± posterior uncertainty					
2018/2/15 6:00	2293.0	2239.4	0.977	±	0.174	1.012	±	0.030
2018/2/22 6:00	1185.5	1104.7	0.932	±	0.336			
2018/3/5 6:00	2784.1	2750.6	0.988	±	0.143			
2018/3/12 6:00	1420.0	1454.0	1.024	±	0.281			
2018/3/17 6:00	2665.5	2750.0	1.032	±	0.149			
2018/3/23 6:00	2045.0	2146.6	1.050	±	0.195			
2018/3/24 6:00	1503.4	1582.9	1.053	±	0.265			
2018/4/16 6:00	3479.1	3520.4	1.012	±	0.115			
2018/4/23 6:00	1239.4	1268.0	1.023	±	0.321			
2018/5/5 6:00	1160.8	1176.8	1.014	±	0.343			
2018/5/28 6:00	2714.2	2698.1	0.994	±	0.147			
2018/5/29 6:00	1577.2	1548.4	0.982	±	0.253			
2018/6/4 6:00	1404.8	1412.3	1.005	±	0.284			
2018/9/14 6:00	1531.8	1517.0	0.990	±	0.260			
2018/10/1 6:00	3215.3	3248.6	1.010	±	0.124			
2018/10/2 6:00	1290.8	1333.5	1.033	±	0.309			
2018/10/13 6:00	2699.3	2708.2	1.003	±	0.148			
2018/10/14 6:00	1228.9	1279.1	1.041	±	0.324			
2018/10/20 6:00	1222.4	1330.3	1.088	±	0.326			
2018/10/26 6:00	1346.1	1349.3	1.002	±	0.296			
2018/10/31 6:00	2617.5	2652.9	1.014	±	0.152			
2018/11/6 6:00	2545.8	2573.9	1.011	±	0.157			
2018/12/31 6:00	1165.0	1173.8	1.008	±	0.342			

Supplementary Table S5: WRF-Chem model configuration for Ulaanbaatar in the forward model.

Version of WRF-Chem	WRF-Chem 4.0[1]
Dynamic core of WRF	Advanced Research WRF (ARW)
Horizontal resolution	9 km (255×145)
Number of vertical layers	35
Initial and lateral boundary condition (Meteorology)	NCEP final reanalysis data (NCEP/FNL)[2]
Initial and lateral boundary condition (CO_2)	JENA_s04oc (CO_2)[3]
Anthropogenic CO_2 emission data	estimated a priori total CO_2 emissions from Ulaanbaatar using the ratio of GDP between 2015 and 2018, whose a priori data was estimated by the project of the Japanese Ministry of Environment as "Project on Development of Innovative Green Technology and MRV Method for JCM in Mongolia (JCM)."
CO_2 emissions by biomass burning	FINN version 1.5[4]
CO_2 emissions by biological resources	MEGAN[5]
Targeting term for forward simulation	1st Jan 2018–31st Dec 2018 (365 days)
Time step	27 seconds
Cloud microphysical process	Morrison 2-mom scheme[6]
Shortwave radiation process	Goddard scheme[7]
Longwave radiation process	RRTMG scheme[8]
Land surface model (LSM)	Noah-MP LSM[9,10,11]
Planetary boundary layer scheme	Mellor, Yamada, Janjic (MYJ) scheme[12]
Chemical transport model	Greenhouse Gas tracer[1]
Photolysis scheme	Fast-J photolysis scheme (CO_2)[13]
CO_2 flux model	VPRM[14,15]

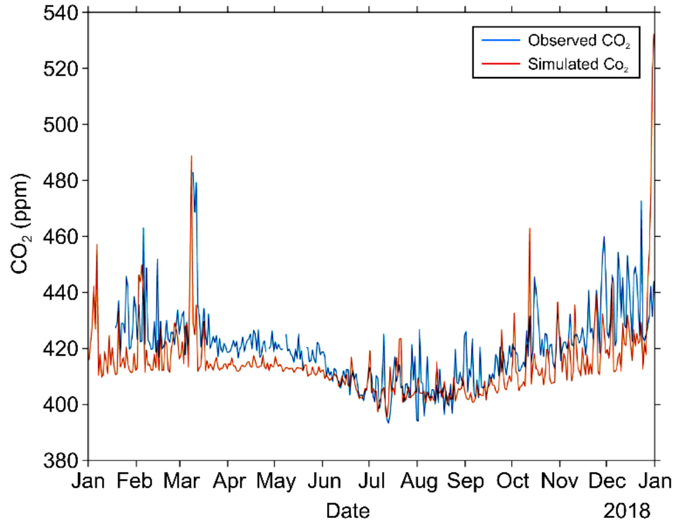

Supplementary Fig. S1: Time series of CO_2 concentrations at 14:00 (local time) in 2018.

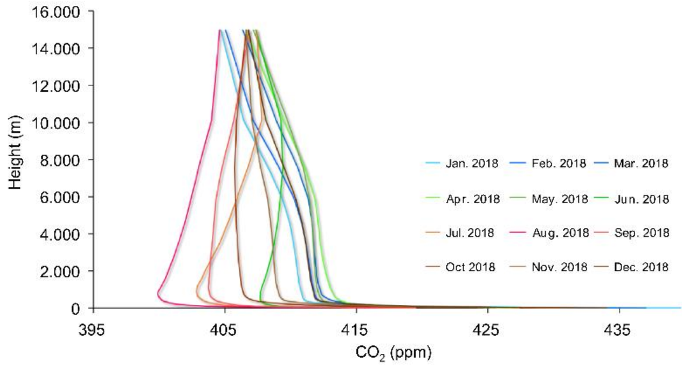

Supplementary Fig. S2: Vertical profiles of CO_2 from WRF-Chem results.

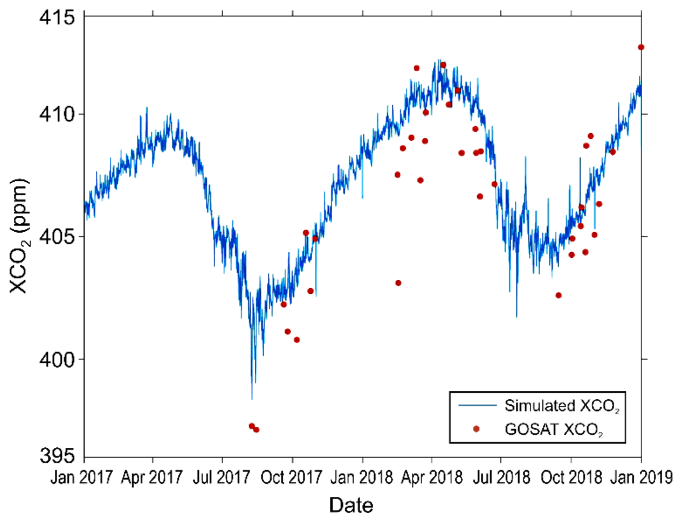

Supplementary Fig. S3: Comparison between simulated XCO_2 (via the WRF-Chem model) and XCO_2 observations from GOSAT at Mongolia 6 station, corresponding to Ulaanbaatar's city centre. WRF-Chem was run between January 1, 2017 and December 31, 2018; GOSAT XCO_2 data are plotted between August 1, 2017 and June 11, 2019.

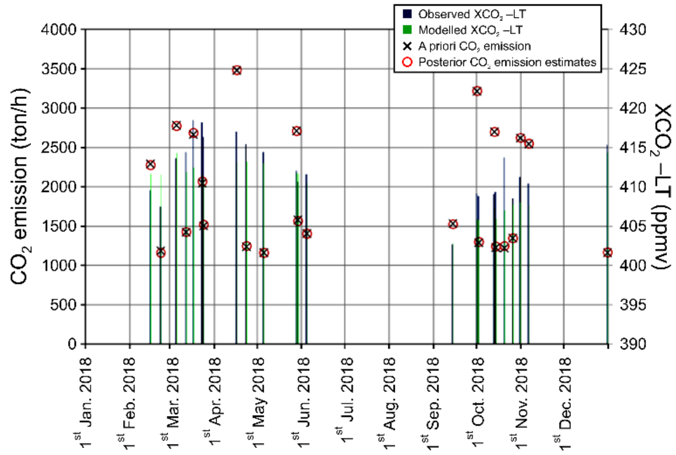

Supplementary Fig. S4: Inversion results of different combination scenarios for sensitivity of CO_2 emission uncertainty and XCO_2 standard deviation. A priori CO_2 emission uncertainty and XCO_2LT standard deviation of 200 ton/h and 2 ppm (case a).

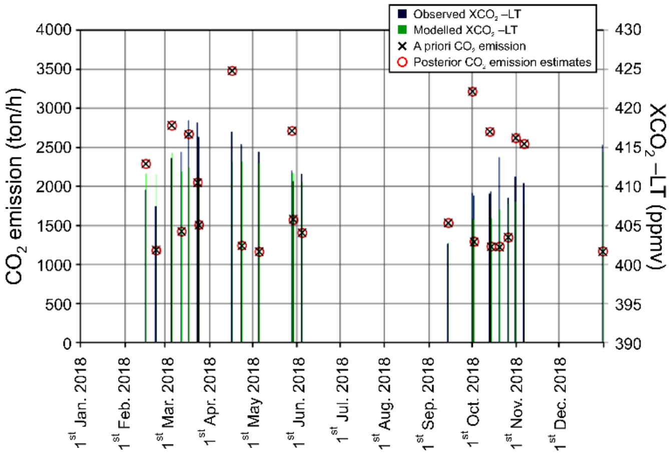

Supplementary Fig. S5: Inversion results of different combination scenarios for sensitivity of CO_2 emission uncertainty and XCO_2 standard deviation. A priori CO_2 emission uncertainty and XCO_2LT standard deviation of 200 ton/h and 4 ppm (case b).

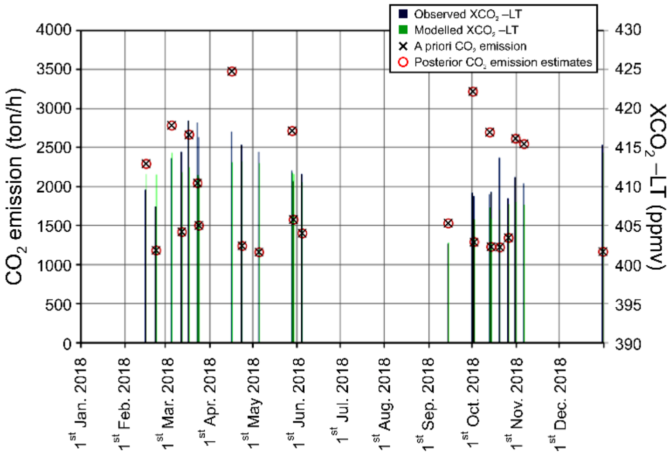

Supplementary Fig. S6: Inversion results of different combination scenarios for sensitivity of CO_2 emission uncertainty and XCO_2 standard deviation. A priori CO_2 emission uncertainty and XCO_2LT standard deviation of 200 ton/h and 8 ppm (case c).

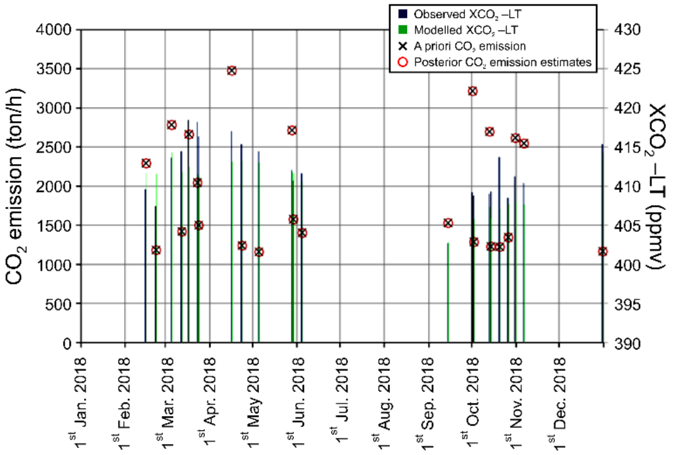

Supplementary Fig. S7: Inversion results of different combination scenarios for sensitivity of CO_2 emission uncertainty and XCO_2 standard deviation. A priori CO_2 emission uncertainty and XCO_2LT standard deviation of 200 ton/h and 16 ppm (case d).

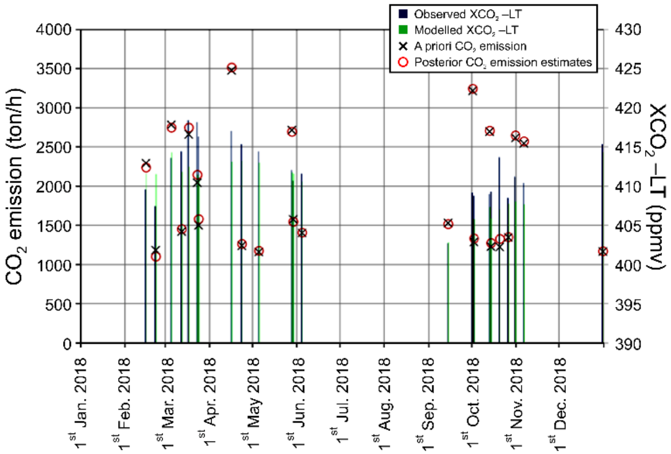

Supplementary Fig. S8: Inversion results of different combination scenarios for sensitivity of CO_2 emission uncertainty and XCO_2 standard deviation. A priori CO_2 emission uncertainty and XCO_2LT standard deviation of 400 ton/h and 2 ppm (case e).

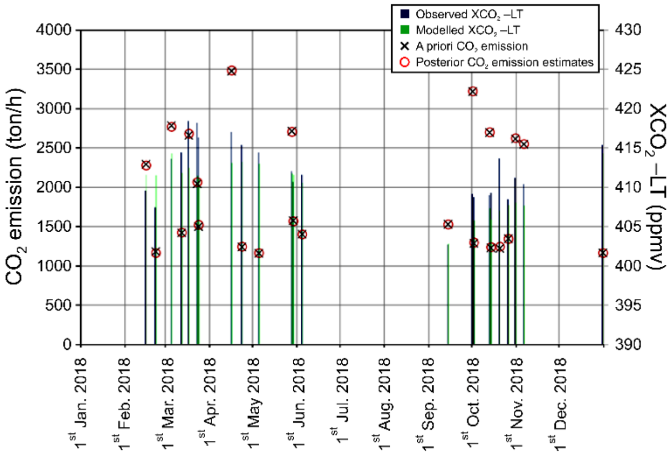

Supplementary Fig. S9: Inversion results of different combination scenarios for sensitivity of CO_2 emission uncertainty and XCO_2 standard deviation. A priori CO_2 emission uncertainty and XCO_2LT standard deviation of 400 ton/h and 4 ppm (case f).

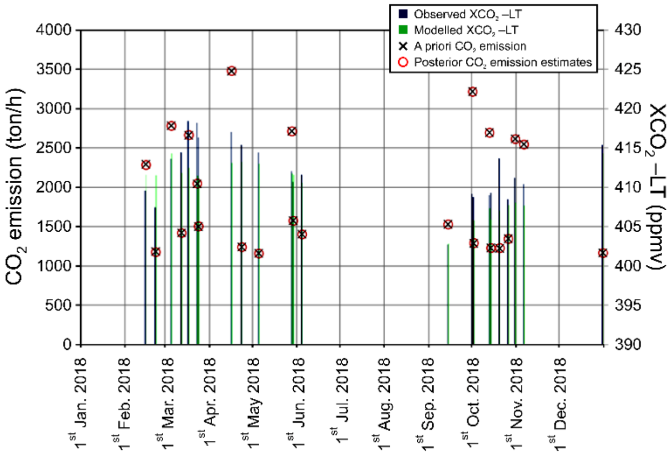

Supplementary Fig. S10: Inversion results of different combination scenarios for sensitivity of CO_2 emission uncertainty and XCO_2 standard deviation. A priori CO_2 emission uncertainty and XCO_2LT standard deviation of 400 ton/h and 8 ppm (case g).

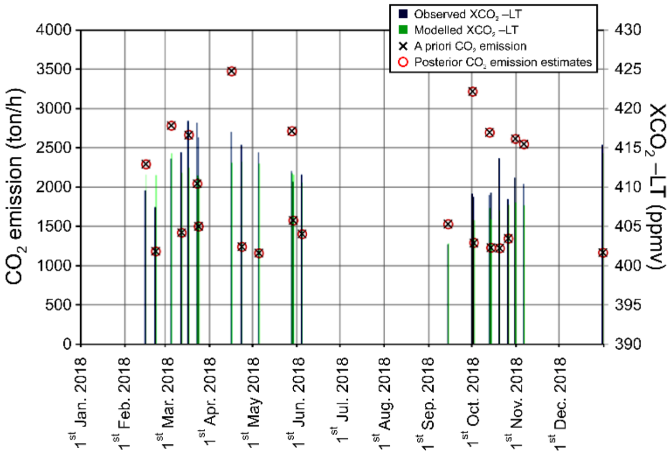

Supplementary Fig. S11: Inversion results of different combination scenarios for sensitivity of CO_2 emission uncertainty and XCO_2 standard deviation. A priori CO_2 emission uncertainty and XCO_2LT standard deviation of 400 ton/h and 16 ppm (case h).

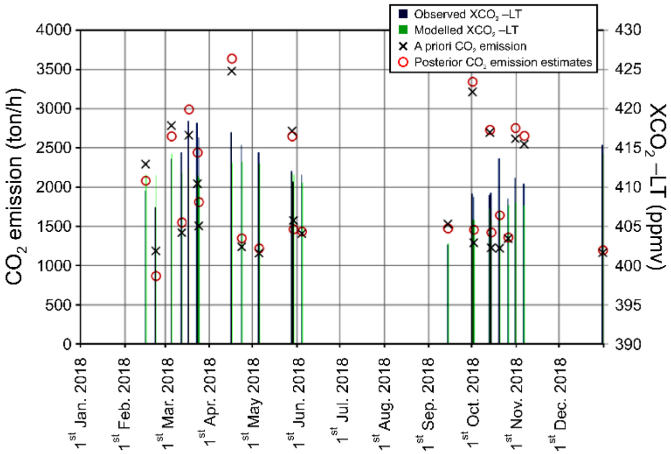

Supplementary Fig. S12: Inversion results of different combination scenarios for sensitivity of CO_2 emission uncertainty and XCO_2 standard deviation. A priori CO_2 emission uncertainty and XCO_2LT standard deviation of 800 ton/h and 2 ppm (case i).

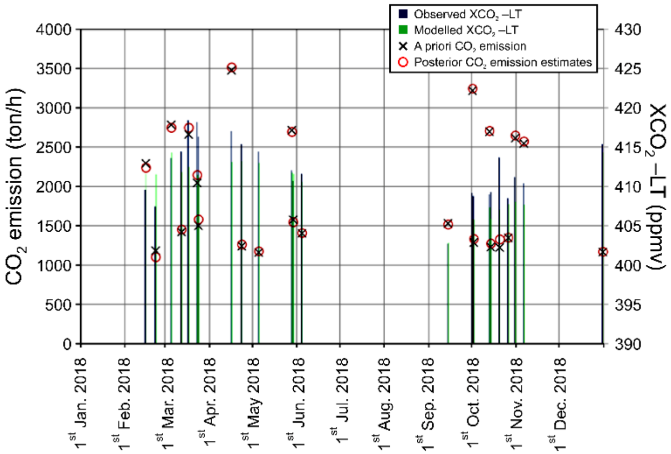

Supplementary Fig. S13: Inversion results of different combination scenarios for sensitivity of CO_2 emission uncertainty and XCO_2 standard deviation. A priori CO_2 emission uncertainty and XCO_2LT standard deviation of 800 ton/h and 4 ppm (case j).

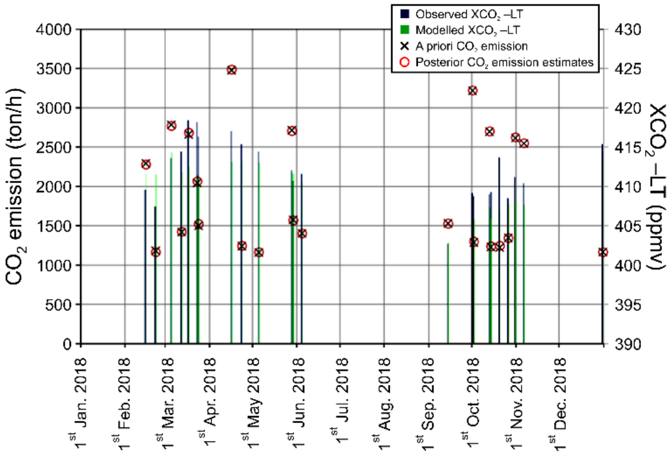

Supplementary Fig. S14: Inversion results of different combination scenarios for sensitivity of CO_2 emission uncertainty and XCO_2 standard deviation. A priori CO_2 emission uncertainty and XCO_2LT standard deviation of 800 ton/h and 8 ppm (case k).

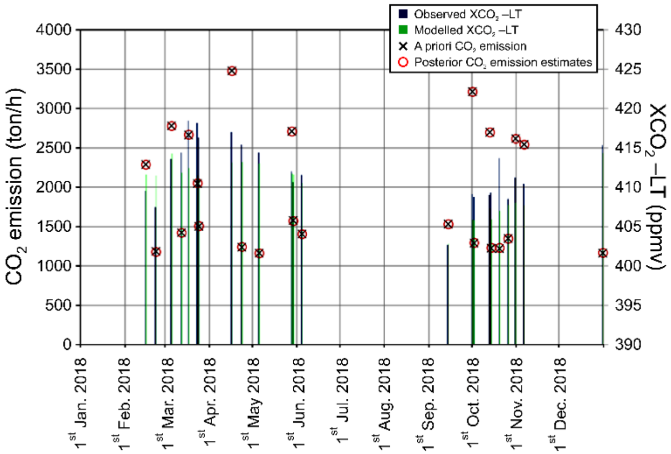

Supplementary Fig. S15: Inversion results of different combination scenarios for sensitivity of CO_2 emission uncertainty and XCO_2 standard deviation. A priori CO_2 emission uncertainty and XCO_2LT standard deviation of 800 ton/h and 16 ppm (case l).

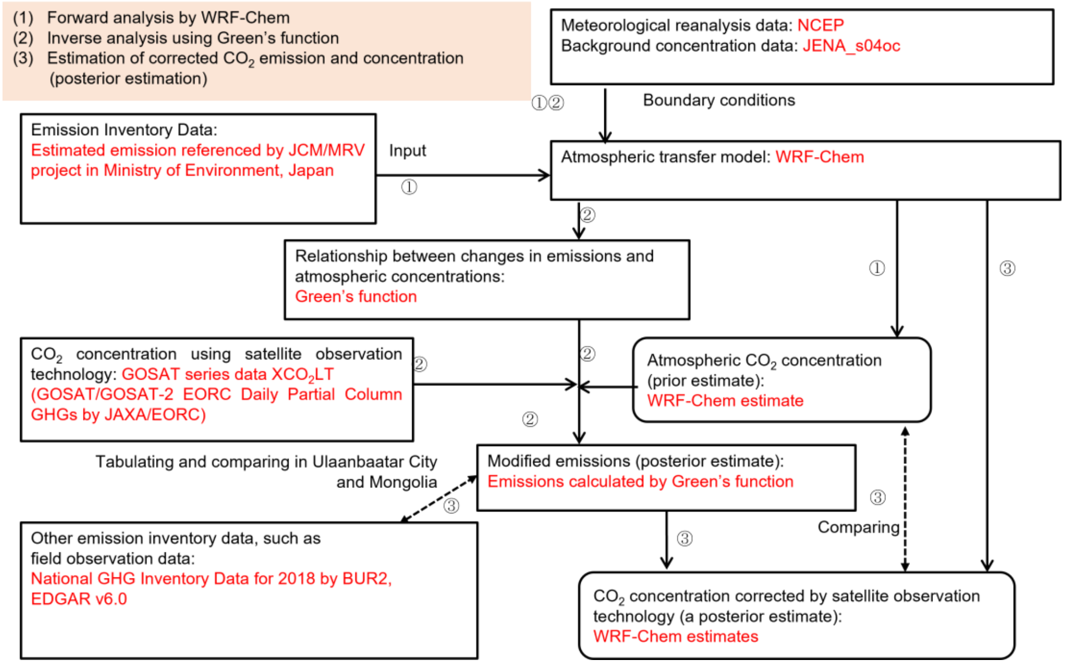

Supplementary Fig. S16: Steps for the inverse analysis of CO_2 emissions from CO_2 concentrations using satellite observation technology in Ulaanbaatar and its suburbs.

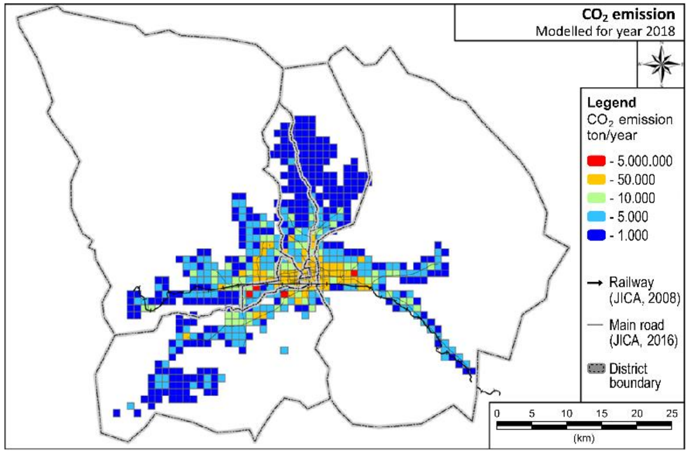

Supplementary Fig. S17: A priori CO_2 emission distribution in Ulaanbaatar.

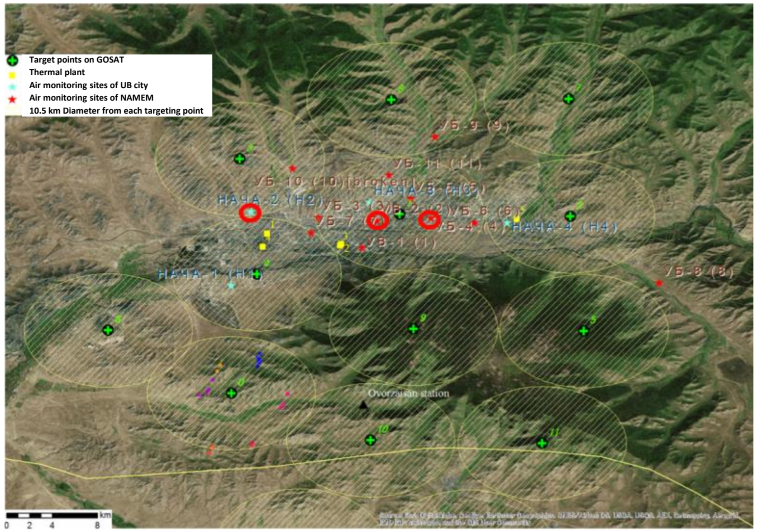

Supplementary Fig. S18: Geographical location of air pollutant monitoring sites in Ulaanbaatar (blue star) and NAMEM (red star). Monitoring sites of CO_2 concentrations used in this study for the estimation of hourly CO_2 emissions (red bold circles), which are NACHA-2 (H2), UB-2 (2), and UB-4 (4).

The map was drawn using the software ArcGIS Desktop: Release 10.2.2 (https://www.esri.com/en-us/arcgis/products/arcgis-for-personal-use/overview). Spatial locations of the monitoring sites and plants were provided by Information and Research Institute of Meteorology, Hydrology and Environment, Mongolia. GOSAT observed points were download by GOSAT/GOSAT-2 EORC Daily Partial Column GHGs (https://www.eorc.jaxa.jp/GOSAT/GPCG/index_GOSAT.html)

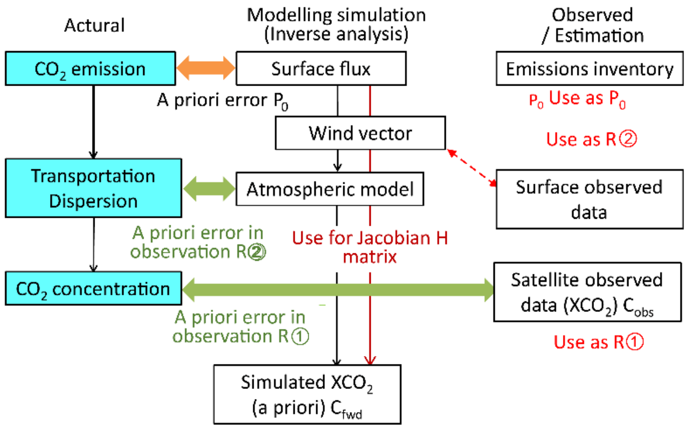

Supplementary Fig. S19: Uncertainties in the inverse modelling framework.

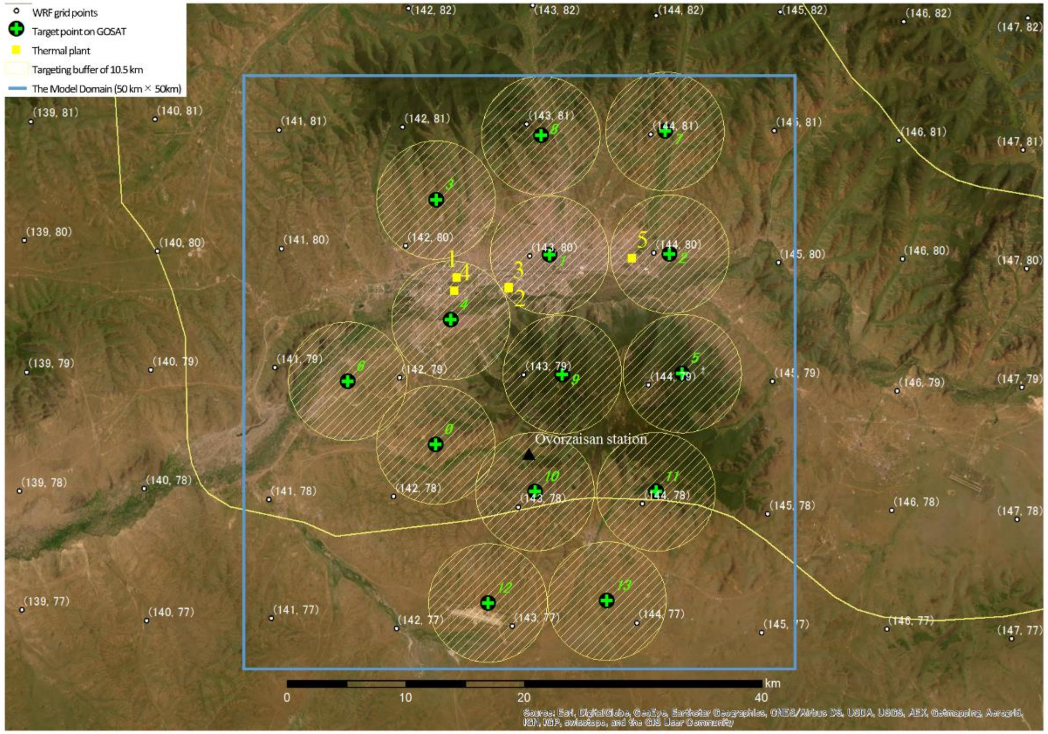

Supplementary Fig. S20: Geographical location of the study area (blue square indicates the model domain) and grid points on the WRF-Chem model.

The map was drawn using the software ArcGIS Desktop: Release 10.2.2 (https://www.esri.com/en-us/arcgis/products/arcgis-for-personal-use/overview). GOSAT observed points were download by GOSAT/GOSAT-2 EORC Daily Partial Column GHGs (https://www.eorc.jaxa.jp/GOSAT/GPCG/index_GOSAT.html). Spatial locations of plants were provided by Information and Research Institute of Meteorology, Hydrology and Environment, Mongolia. Spatial locations of the monitoring sites and the WRF grids were plotted by authors.

Supplementary Note 1. Comparison between modelled and observed CO_2

In situ atmospheric CO_2 was measured on the roof of the research institute in Ulaanbaatar. The air inlet mouth was installed at a height of approximately 10 m. Data were recorded using an ultraportable greenhouse gas analyser (UGGA, Los Gatos Research), which was calibrated using a standard gas cylinder. A standard deviation of 0.07 ppm was evaluated over 2 min. Supplementary Fig. S1 shows a comparison between the modelled and observed CO_2 concentration data at 14:00 (local time) in 2018. Supplementary Table 2 contains comparison statistics for these data.

Supplementary Note 2. XCO_2LT enhancement over Ulaanbaatar

We considered the availability of lower-troposphere (LT) concentration data from the GOSAT-based 2-layer analysis product by the Japan Aerospace Exploration Agency/Earth Observation Research Center (JAXA/EORC). The LT partial column is defined as the layer in which the atmospheric pressure ranges from 0.6 to 1 P_{surf}, which typically includes all the air from the ground to an altitude of approximately 4 km. Although validation for the retrieved LT data was only performed via spiral flights over Nevada (USA)[19], we argue that LT observations would show higher GHG emissions than the whole-column average value. We reported the seasonal variation in LT concentrations and noted that the relatively high wind speeds and slow vertical mixing in the upper troposphere function as a relatively small seasonal cycle in the upper troposphere. We took the CO_2 enhancement in the LT as an indicator of normalized CO_2 in the LT, which is calculated as[17]:

$$\Delta XCO_2\ (d)^{LT}{}_{ave} = \sum_{d}^{N} \frac{XCO_2(d,k)^{LT} - XCO_2(m)^{UT}_{average}}{N} \qquad (3)$$

where $XCO_2(m)^{UT}_{average}$, d, k, and N denote the XCO_2UT monthly average over Ulaanbaatar calculated to reduce the annual CO_2 trend and growth, the observation day, the observation point, and the total number of successfully retrieved data for the city, respectively. We only considered observation days during which data from at least 10 of the 14 observation points were retrieved.

Supplementary Note 3. Targeted hours for emissions in Green's function

Equations [1] and [2] were applied to a 50 km × 50 km model domain in and around Ulaanbaatar in 2018 to calculate the a posteriori estimate of GHG emissions (Supplementary Fig. S20). We used XCO_2LT concentration data from GOSAT/GOSAT-2 EORC Daily Partial Column GHGs[17,18]. CO_2 emissions values in Green's function correspond to hourly averaged emissions within the model domain over 24 h from GOSAT's observation time. We calculated the a posteriori emissions when, in the model domain, data from at least 70% of the GOSAT observation points were retrieved (i.e., at least 10 out of 14 points). Finally, we calculated the average ratio of a posteriori CO_2 emissions to a priori CO_2 emissions within the model domain over a year.

Supplementary Note 4. Sensitivity analysis to assess the robustness of the top-down estimate

We performed a sensitivity analysis to assess the effect of a priori emissions and GOSAT XCO_2 values on the top-down estimation results. Supplementary Figs. S4–S15 display an overview of different combination scenarios used to investigate the effect of the standard deviation of XCO_2LT values at 2–16 ppmv and a priori emission uncertainties of 200, 400, and 800 ton h^{-1}(13–51%). Supplementary Table S3 also shows results with a priori emission uncertainties of 200, 400, and 800 ton h^{-1}(13–51%) and a standard deviation of XCO_2LT set to 2 ppmv.

Supplementary Note 5. Hourly input data computation of a priori CO_2 emissions

The forward model in this study relied on hourly input data of a priori CO_2 emissions in WRF-Chem. CO coemitted with CO_2 mainly from combustion indicates a positive correlation, particularly when combustion is the dominant source of CO_2[20,21].

$$e_t = \frac{q(t)}{S(m)} E_m \qquad [1]$$

where e_t represents the CO_2 emissions at time t, $q(t)$ represents the CO concentrations at time t, $S(m)$ is the total amount of CO during month m, $q(t)/S(m)$ is the ratio of CO concentrations at time t to the total CO in month m, and E_m is the total CO_2 emission in month m. In [1], q(t) is calculated as follows:

$$S(m) = \sum_1^d q(t) \qquad [2]$$

where d represents the total hours in a given month m (e.g., in August, d = 744 h).

E_m in [1] is the calculated monthly CO_2 emission (1 km resolution) for Ulaanbaatar in 2018. We used CO concentrations as hourly observed data provided by the research institute in Mongolia. Note that a priori CO_2 emissions for Ulaanbaatar were spatially calculated based on data of CO_2 emissions from thermal power plants (4 sites), automobiles, gers (small household stoves), heat-only boilers, and Coal Fired Water Heaters in 2015, calculated by the Japanese Ministry of Environment project within the "Project on Development of Innovative Green Technology and MRV Method for JCM in Mongolia (JCM)". We calculated a priori total CO_2 emissions from Ulaanbaatar in 2018 using the ratio of GDP[16] between 2015 and 2018 (Supplementary Table S1, Supplementary Fig. S17).

For the calculation of hourly CO_2 emissions from gers, we used CO concentration observation data at the NACHA-2 station, which is managed by the Ulaanbaatar city administration. For the calculation of hourly emissions from roads, CO concentration data from UB-2, which is managed by NAMEM, were used. In the case data from UB-2 were missing, data from UB-4 were used (Supplementary Fig. S18).

References

1. Beck, V. et al. WRF-Chem simulations in the Amazon region during wet and dry season transitions: evaluation of methane models and wetland inundation maps. *Atmos. Chem. Phys.* **13**, 7961–7982 (2013).
2. National Center for Atmospheric Research. NCEP FNL Operational Model Global Tropospheric Analyses, continuing from July 1999. https://rda.ucar.edu/datasets/ds083.2/ (retrieved on August 3, 2022).
3. Max Planck Institute for Biogeochemistry. Atmospheric CO_2 Inversion s10oc_v2021 Atmospheric CO_2 fields. https://www.bgc-jena.mpg.de/CarboScope/?ID=s (retrieved August 10, 2022).
4. Wiedinmyer, C. et al. A high resolution global model to estimate the emissions from open burning. *Geosci. Model Dev.* **4**, 625–641 (2011).
5. Henrot, A.-J. et al. Implementation of the MEGAN (v2.1) biogenic emission model in the ECHAM6-HAMMOZ chemistry climate model. *Geosci. Model Dev.* **10**, 903–926 (2017).
6. Morrison, H., Thompson, G. & Tatarskii, V. Impact of Cloud Microphysics on the Development of Trailing Stratiform Precipitation in a Simulated Squall Line: Comparison of One- and Two-Moment Schemes. *Mon. Weather Rev.* **137**, 991–1007 (2009).
7. Tao, W.-K. et al. Microphysics, radiation and surface processes in the Goddard Cumulus Ensemble (GCE) model. *Meteorol. Atmos. Phys.* **82**, 97137 (2003).
8. Iacono, M.J. et al. Radiative forcing by long-lived greenhouse gases: calculations with the AER radiative transfer models. *J. Geophys. Res. Atmos*. **113**, D13103 (2008).
9. Yang, Z.-L. et al. The community Noah land surface model with multiparameterization options (Noah-MP): 2. Evaluation over global river basins. *J. Geophys. Res.* **116**, D12110 (2011).
10. Niu, G.-Y. et al. The community Noah land surface model with multiparameterization options (Noah-MP): 1. Model description and evaluation with local-scale measurements. *J. Geophys. Res.* **116**, D12109 (2011).
11. Barlage, M. et al. The effect of groundwater interaction in North American regional climate simulations with WRF/Noah-MP. *Clim. Change* **129**, 485–498 (2015).
12. Janjic, Z. Nonsingular implementation of the Mellor–Yamada level 2.5 scheme in the NCEP mesomodel. NCEP Office Note No. 437, 60 (2002).
13. Wild, O., Zhu, X., & Prather, M. J. Fast-J: Accurate simulation of in- and below-cloud photolysis in Global Chemical Models. *J. Atmos. Chem.* **37**, 245–282 (2000).
14. Ahmadov, R. et al. Mesoscale covariance of transport and CO_2 fluxes: Evidence from observations and simulations using the WRF-VPRM coupled atmosphere–biosphere model. *J. Geophys. Res.* **112**, D22107 (2007).
15. Mahadevan, P. et al. A satellite-based biosphere parameterization for net ecosystem CO_2 exchange: Vegetation Photosynthesis and Respiration Model (VPRM). *Global Biogeochem. Cycles* **22**, GB2005 (2008).
16. World Bank. World Bank national accounts data, and OECD National Accounts data files (constant LCU). https://data.worldbank.org/indicator/NY.GDP.MKTP.KN?locations=MN (2022).
17. Kuze, A. et al. Examining partial-column density retrieval of lower-tropospheric CO_2 from GOSAT

target observations over global megacities. *Remote Sens. Environ.* **273**, 112966 (2022).

18. Kikuchi, N., Yoshida, Y., Uchino, O., Morino, I. & Yokota, T. An advanced retrieval algorithm for greenhouse gases using polarization information measured by GOSAT TANSO-FTS SWIR I: Simulation study. *J. Geophys. Res.* **121**, 13129–13157 (2016).
19. Tanaka, T. et al. Two-year comparison of airborne measurements of CO2 and CH4 With GOSAT at railroad Valley, Nevada. IEEE Trans. *Geosci. Remote Sens.* **54**, 4367–4375 (2016).
20. Wang, Y., Munger, J. W., Xu, S., McElroy, M. B., Hao, J., Nielsen, C. P., & Ma, H. CO2 and its correlation with CO at a rural site near Beijing: implications for combustion efficiency in China. *Atmospheric Chemistry and Physics*. **10** (18), 8881-8897 (2010).
21. Yuan, L., & Smith, A. C. CO and CO2 emissions from spontaneous heating of coal under different ventilation rates. *International Journal of Coal Geology.* **88** (1), 24-30 (2011).
